# A Practical Treatise on the Diseases of Children

**Published:** 1843-12-23

**Authors:** 


### BIBLIOGRAPHICAL NOTICES.

*A Practical Treatise on the Diseases of Children.* By D. Francis Condie, M. D., etc. etc. Philadelphia: Lea & Blanchard. 1844. 8vo. pp. 651.

The object of the author in the present treatise is, "to present a full and connected view of the actual state of the pathology and therapeutics" of the diseases which occur between birth and puberty. The pathology of infantile affections has been of late years much extended and elucidated, and simpler and more certain therapeutics have been the consequence. The older works on this subject are, therefore, deficient in many points of importance, and are very imperfect guides for the student. The foreign treatises, many of which are excellent, are defective, so far as regards the forms of disease peculiar to our climate. A practical work on the Diseases of Children was, therefore, much needed in this country, and the present one is intended to fulfil this indication. Let us see how successfully this has been done.

Whilst the author has liberally and judiciously appropriated the labours and opinions of others, this has generally been done when their views were confirmed by his own observation and experience, which have been "acquired during a long and somewhat extensive practice, and under circumstances peculiarly well adapted for the clinical study of the diseases of early life." In a few instances only, where the authority was high and unquestionable, has this rule been deviated from. On those points where decided difference of opinion prevails in the profession, our author has adopted those views which coincided with the results of his own observation, giving, at the same time, when the importance of the subject demanded it, "a fair exposition of the views of others in relation to it."

The work is divided into two parts; the first treats of infantile hygienics and general pathology, and the second is devoted to special pathology, both internal and external.

The nature of the work necessarily precludes any extended critical or analytical notice. The author is evidently a man of considerable practical familiarity with his subject, and at the same time a laborious student; two essential requisites in a writer. He has apparently thought much and well on the various points he treats of. His pathological views generally please us; with extensive knowledge, there is much judgment and reflection. The same may be said of his therapeutics. The character of the work is far above that of a summary of the actual condition of infantile pathology. It is an excellent *Practical Treatise on the Diseases of Children*, and a very safe guide to the juvenile practitioner and student. The limited space necessarily precluded much detail; but our author's style is condensed, and the retrenchments have been made commonly at the expense of the theoretical portions of the work. We welcome its appearance, and have no doubt that it will assume and maintain its rank amongst our standard medical books. With this opinion we will venture the following critical remarks.

The pretensions of the work, however justly, are rather too warmly stated in the Preface, on the score of taste. The old adage of "good wine needing no bush," is applicable in the present instance. An author, aware of the difficulties he has encountered, full of his subject, and frequently feeling his superiority, is disposed to make the public a participator in his feelings; but this is rarely reciprocal, the latter being cool and unsympathizing. We do not understand on what just pathological grounds Jaundice and Cyanosis are ranked among skin-diseases. The exploded orthography of scrofula (scrophula) is disinterred, with what view we cannot comprehend, the derivation being from *scrofa;* and nutritious is spelt *nutricious*, on what authority we are at a loss to say. At p. 144 we notice that "a few grains of aqua camphorata or spirits of turpentine" are ordered. The numerous references in the body of the page are, we think, disfiguring and unnecessary; many might be omitted with advantage, and the remainder incorporated with the text. These may be unimportant matters, but we are unwilling to see any blemishes upon so excellent and timely a work.

